# Tumors of the Mouth and Jaws

**Published:** 1888-04

**Authors:** A. E. Baldwin


ARTICLE VI.

### TUMORS OF THE MOUTH AND JAWS.

A. E. BALDWIN, M. D., D. D. S.

[Read before the Chicago Dental Society, February, 1888.]

As stated by the speaker a few months ago, he had labored under the delusion of being allowed to choose his own subject for his paper, and has chosen one, and worked upon it a long time. The subject assigned in this instance is one of which the writer has but little knowledge. But realizing that many come to the society to hear papers on the listed subject, and possibly loaded with much that is of interest in the discussion of the same, he determined to adhere to the adopted plan, and write on the subject assigned, hoping that at another time he may be allowed some choice. In advance he wants to state that this paper will consist largely of the thoughts of others, given as briefly as possible, and the original matter will be infinitesimally small, save in the latter part of the paper, where the writer has to strain a point to bring what he says under the head of "Tumors of the Mouth and Jaws."

The general term "Tumor" covers the ground of any swelling or abnormality in excess of normal size in any tissue. However, the term is generally used when the enlargement is accompanied by pathological conditions. The tumors of which we speak are in the hard or bony parts, and in the soft or fleshy tissues. We may with propriety divide them into two classes, those produced by local causes or conditions, and the other by outside or foreign influences.

The first class are of necessity benign in their character, and the second class may be either benign or malignant. In the proper diagnosis of these growths or developments it has long been a rule in surgery that the normal tissue must be understood; hence the necessity of every dentist being a careful and observant examiner in every mouth—not only of the tissue, the treatment of which calls the patient to him, but of all the tissues in the range of vision, so as to be able to recognize the normal condition. For if the examiner is ignorant of the normal he cannot expect to understand the abnormal.

When the natural is clearly understood, then the diagnosis of the unhealthy parts is made easy by the process called exclusion, and oftentimes a diagnosis may be arrived at in this way—that would be impossible in any other way. The writer does not wish to be understood as saying that all conditions are easy of diagnosis, for often it baffles the most skillful; and in this matter, as in all others pertaining to our specialty, those succeed best who use judgment and thought as to cause and effect, and he thinks the tendency of all is to examine to find proofs of substantiation of preconceived notions. The true scientist is the one who disarms himself of this, and examines with an eye only to the histological and pathological conditions; and as a rule, when the condition and the cause is understood the treatment suggests itself.

In the first division of tumors we may have exostosis—its name describes its nature; Sarcoma—this quite rare, and then the most common, cystic tumors of the jaws, many times produced by morbid enlargement or dilatation of the dental follicle. These occur in the alveolar ridge, and sometimes attain a great size. The contents are usually liquid, or gelatinous, and occasionally contain rudimentary teeth.

There is another growth, which is developed from the bony or the soft tissues, or from both, with the name Epulis. Virchow suggests that the nature of the growth be added to the term epulis, as epulis sarcomatosa, e. myxomatosa. The greater number belong to the sarcomas, and vary in their character all the way from the round cell sarcoma to the hard, firm fibro-sarcoma. This epulous growth may be from the osseous, or soft tissues, or more often having its origin from both. The growths are largely made up of giant cells, and usually occur in young subjects. The growth is varied—sometimes very rapid, and at other times very slow. They are very vascular, and this gives them a darkened color. As a rule, they are non-malignant, but the tendency is strong to a recurrence and spreading. When one determines to remove one of these growths care should be taken to remove all, and a rule among surgeons should be remembered, that is, cut away freely till you are positive nothing but healthy tissue remains; and in these cases usually a portion of the bone must be removed. The writer thinks that a fear of too much removal is at the bottom of many of the recurrences, and thinks that very few, if any, of them where everything is removed that pertains to the tumor.

Among the tumors in the soft parts are principally the angiomata—composed largely of vascular tissue—and are often called erectile tumors, and these vary in size at different times; another form, and more rare, are the lymphangiomata, the nature of which is indicated by the name.

Later in life we have *sarcoma* growths, and what has been said in regard to epulis applies largely to this. Carcinoma attacks (in the form of epithelioma) the soft tissues, beginning usually as a small nodule, or circumscribed hard, grayish infiltration of the mucous membrane. Then, we may have tumors as a result of the distension of glands from aceumulated and retained secretions. The treatment of these is usually very simple, viz: to open the ducts and allow the contents to escape. Sometimes a lymphatic gland, by inflammatory dilation, gives rise to cystic tumors, known as cystic hygromata; and then various others in mucous structurea in different parts of the mouth, known under the general term of Ranula.

We should be well assured that we understand fully the nature of the growth, or condition of the abnormal tissue, ere adopting any proceeding toward the eradication of it. The diagnosis can not be made too certain. Sometimes, the writer fears, if this is not remembered, grave mistakes may be made. Where the growth is causing but little disturbance, and developing slowly, or is staticnary, the best treatment is to let it alone. Meddlesome surgery in these cases is often dangerous; provoking, sometimes, a much more rapid recurrence.

In malignant cases, where the growth is of recent origin, or easily bounded, and the general condition of the patient is favorable (and here very much depends upon this general condition)—in such cases an operation should be urged, and the operator must remove freely. In case he makes any mistake, let it be on the side of removing too much rather than too little, for if any of the morbid growth remains it will act as a nidus for future growths, which are usually much more rapid than the original.

There remain many things to say in regard to the general subject—a much longer paper might profitably be written on only one class of tumors—but the writer has spent the time necessary to open quite a field for thought and discussion, and if any entertain dinerent opinions than those expressed, the writer most cordially invites them to express them, and hopes a more general knowledge of this subject may be the result.

Before closing this paper he wishes to mention a condition that, perhaps, properly belongs here, but hardly in the pure field of tumors, being, however, closely allied, viz: tumors resulting in a condition known as alveolar abscess. From the time the writer began to think on the subject of dentistry till now, this subject of abscesses, their causes and treatment, has had a special interest to him, and he thinks that he has never heard anyone speak of a case where, when one had treated, and then filled the roots of a tooth abscessed, if trouble recurred the idea was not conveyed that the recurrence was a result of imperfect work, and where our friends, who live among micro-organisms, would shake their heads and say, these canals were not made aseptic, or that some of the micro-organisms got away into pastures new, etc., etc. He was struck with the fact that all seem to agree that when the death and removal of the pulp was immediately followed by root-filling, that trouble seldom, if ever, occurred. But if the tooth pulp has been dead some time most dentists seem afraid to immediately fill, claiming they are unsuccessful in so doing—such cases giving trouble. Many claim this as a practical illustration that septic germs have been carried through the apical foramen, and its consequent ills are thus accounted for. You all know how enthusiastic these micro-organism enthusiasts can beeome, crying Eureka! in every gathering, and from their very assurance causing many to say, Amen! Now, the writer believes that in the main these claims are not true, and challenges them to proof. He has in a simple way experimented sufficiently to feel assured that in many cases where the pulp has been dead some time there has been inflammation of the pericementum sufficient to produce an abscess, and that in very many of these cases there has resulted, from the pressure and infiltration of the purulent matter, necrosis of a portion of the alveolus, generally very circumscribed, sometimes quite extensive.

Now, it may be expected that where this is the case the dead bone will give trouble sooner or later, and usually very soon—when the opening through the tooth is stopped, however perfect the root filling may be; of course, if there is a fistulous opening the change will not be noticed. Now, he believes that when these roots are cleaned out and dried thoroughly, and when filled, no trouble will occur from anything within the root or the tooth, but if care and thoroughness obtains we can tell by inflammations following the work that the trouble is outside of the tooth, and probably there is necrosed bone present. He can illustrate by two typical cases. Case 1: Patient had had a tooth filled, with a resulting abscess; three weeks later came to him; he found a fistulous tract; opened the tooth and carefully cleaned and dried the canals, and filled them and the tooth at the same sitting; four weeks later patient came again and said fistula had not closed yet; injected cocaine, cut down and removed a scale of necrosed bone; the wound healed kindly, and now, six months later, shows no sign but perfect health.

Case 2: Patient came with only roots of central and lateral left superior incisors remaining; they had abscessed twice, no fistulæ; wished to have them extracted, and, as is the writer's custom, he persuaded the patient to let him fill the roots first, and wait and see the results; patient reported forty-eight hours later with every sign of beginning abscess; he then extracted, and, as usual, found the roots perfectly filled, but on probing into the sockets dead bone was found, and, no doubt, caused the trouble. Here the roots spoken of had been filled forty-eight hours when extracted. Many similar cases might be cited, but this illustrates what is claimed: that, in his belief, in every case that can be dried thoroughly and perfectly filled, no trouble will arise from any condition within the tooth, and that in those cases where trouble arises it is from outside the tooth, and in many, or most cases, is caused by the necrosed bone; and the proper thing to do is to open down upon it and treat from the outside, and bur out the necrosed bone. Some members of our specialty seem inclined to make of this quite a surgical operation, but the writer thinks no professional duty is more simple He injects about three or four minims of a ten-per-cent. solution of cocaine then makes an incision down to the part, and with a fair-sized bur cuts and then washes out the dead bone. This is all done with probably a mouthful of micro-organism, and usually one operation only is needed.—*The Dental Review*.

